# Energy-mediated vs. ammonium-regulated gene expression in the obligate ammonia-oxidizing bacterium, *Nitrosococcus oceani*

**DOI:** 10.3389/fmicb.2013.00277

**Published:** 2013-09-13

**Authors:** Lisa Y. Stein, Mark A. Campbell, Martin G. Klotz

**Affiliations:** ^1^Department of Biological Sciences, University of AlbertaEdmonton, AB, Canada; ^2^Department of Biology, University of North CarolinaCharlotte, Charlotte, NC, USA

**Keywords:** *Nitrosococcus*, ammonia-oxidizing bacteria, ammonium, hydroxylamine, redox, signaling, global gene expression, microarray

## Abstract

Ammonia serves as the source of energy and reductant and as a signaling molecule that regulates gene expression in obligate ammonia-oxidizing chemolithotrophic microorganisms. The gammaproteobacterium, *Nitrosococcus oceani*, was the first obligate ammonia-oxidizer isolated from seawater and is one of the model systems for ammonia chemolithotrophy. We compared global transcriptional responses to ammonium and the catabolic intermediate, hydroxylamine, in ammonium-starved and non-starved cultures of *N. oceani* to discriminate transcriptional effects of ammonium from a change in overall energy and redox status upon catabolite availability. The most highly expressed genes from ammonium- or hydroxylamine-treated relative to starved cells are implicated in catabolic electron flow, carbon fixation, nitrogen assimilation, ribosome structure and stress tolerance. Catabolic inventory-encoding genes, including electron flow-terminating Complexes IV, FoF1 ATPase, transporters, and transcriptional regulators were among the most highly expressed genes in cells exposed only to ammonium relative to starved cells, although the differences compared to steady-state transcript levels were less pronounced. Reduction in steady-state mRNA levels from hydroxylamine-treated relative to starved-cells were less than five-fold. In contrast, several transcripts from ammonium-treated relative to starved cells were significantly less abundant including those for forward Complex I and a gene cluster of cytochrome *c* encoding proteins. Identified uneven steady-state transcript levels of co-expressed clustered genes support previously reported differential regulation at the levels of transcription and transcript stability. Our results differentiated between rapid regulation of core genes upon a change in cellular redox status vs. those responsive to ammonium as a signaling molecule in *N. oceani*, both confirming and extending our knowledge of metabolic modules involved in ammonia chemolithotrophy.

## Introduction

Obligate ammonia-oxidizing chemolithotrophic bacteria (AOB) facilitate key transformations in the global nitrogen cycle that interconnect nitrification, denitrification and ammonification (Klotz and Stein, 2011; Stein and Klotz, 2011; Simon and Klotz, 2013). When facilitated by AOB, all of these processes take place under oxic conditions because the first (mono-oxygenation of ammonia) and last (disposal of electrons) steps of catabolic electron flow require free molecular oxygen. AOB obtain all of their energy and reductant by catabolic oxidation of ammonia to nitrite, the first in the two-step nitrification process historically referred to as nitritation. Thus, AOB consume ammonia and oxygen for both maintenance of proton-motive force and growth via autotrophic chemosynthesis (Arp et al., 2002).

Ammonia oxidation is facilitated by two dedicated enzyme complexes that were once considered unique to AOB (Arp et al., 2002): ammonia monooxygenase (AMO, *amoCAB*, EC:1.14.99.39) and hydroxylamine dehydrogenase (HAO, *haoA*, EC:1.7.2.6). However, in recent times both AMO and HAO complexes have been identified in many diverse genome backgrounds (Klotz and Stein, 2008; Klotz et al., 2008; Sayavedra-Soto et al., 2011; Tavormina et al., 2011, 2013; Coleman et al., 2012; Hanson et al., 2013; Simon and Klotz, 2013). When expressed together in non-chemolithotrophs, such as methane-oxidizing bacteria (MOB; including Proteobacteria and Verrucomicrobia), these two protein complexes facilitate nitritation; however, the electrons extractable from hydroxylamine cannot be used to support growth via chemolithotrophic catabolism (Klotz and Stein, 2011; Stein and Klotz, 2011). Gene expression studies demonstrated that the *haoA*-associated gene *haoB*, adjacent to *haoA* and formerly known as *orf2* (Bergmann et al., 2005), is co-expressed with *haoA* in the genomes of nitritating gammaproteobacterial AOB and MOB (Poret-Peterson et al., 2008; Campbell et al., 2011b). However, in order to utilize the electrons extracted from hydroxylamine during nitritation, AOB but not nitritating MOB employ the cytochrome *c* protein, *c*_M_552 (*cycB*) as a cognizant quinone reductase (Elmore et al., 2007; Kim et al., 2008) that either directly interacts with HAO or obtains electrons indirectly via cytochrome *c*554 (*cycA*) (Hooper et al., 2005; Klotz et al., 2008; Kern et al., 2011; Simon and Klotz, 2013). Earlier results indicated that the *haoA* and *cycAB* genes are under control of different promoters in *N. europaea* (Sayavedra-Soto et al., 1994; Arp et al., 2002; Hommes et al., 2002), however, gammaproteobacterial AOB such as *N. oceani* appear to produce distinct *haoAB, cycAB* and *haoAB-cycAB* transcripts indicating complex regulation similar to what was reported for the regulation of genes in the *amo* gene cluster (El Sheikh and Klotz, 2008; El Sheikh et al., 2008). Cytochrome *c*_M_552 belongs to a large superfamily of membrane-associated cytochrome *c* proteins (NapC/NrfH) that exchange electrons with the quinone/quinol pool (Bergmann et al., 2005; Simon and Klotz, 2013). Generally in bacterial genomes, the *cycB* gene encoding *c*_M_552 is clustered with other genes encoding catalytic periplasmic proteins that facilitate reduction of nitrogen oxides such as nitrate reductase (*nap*), nitrite reductase (*nrf*), and/or homologues of cytochrome *c*554 that function as nitric oxide reductases (Upadhyay et al., 2006; Klotz and Stein, 2011; Simon and Klotz, 2013).

Although the ever-increasing number of sequenced microbial genomes has revealed that *amoCAB, haoAB*, and *cycAB* genes are not unique to AOB (Arp et al., 2007), it appears that ammonia-dependent chemolithotrophy requires the presence and co-expression of all of these gene clusters (Klotz and Stein, 2011). So far, only the gene encoding the small red-copper protein nitrosocyanin, *ncyA*, (Arciero et al., 2002; Basumallick et al., 2005) has been identified in the genomes of all AOB with the exception of the oligotrophic *Nitrosomonas* sp. Is79 strain (Bollmann et al., 2012). The *ncyA* gene is conspicuously absent from other microbes including non-chemolithotrophic nitritating bacteria like the MOB (Campbell et al., 2011b; Klotz and Stein, 2011) and chemolitotrophic nitritating Thaumarchaeota (Walker et al., 2010). As AOB have two nitritation-dependent pathways that result in production of nitric and nitrous oxides, one starting with hydroxylamine oxidation and the other with nitrite reduction, this activity represents a significant drain to the redox status of the bacteria (Stein, 2011). Therefore, electron flow into and out of the quinone pool needs to be tightly regulated, perhaps by employing the product of the *ncyA* gene as a redox sensitive switch that directs electrons extracted by HAO to different acceptor proteins. However, this hypothesis remains to be tested.

To date, there is no clear conceptual understanding of the regulation of the *amoCAB, haoAB, cycAB*, and *ncyA* genes by cellular redox status and/or by general signaling molecules like ammonium (Sayavedra-Soto et al., 1996; Stein et al., 1997; Bollmann et al., 2005; Wei et al., 2006) or nitrogen oxides (Cho et al., 2006; Beyer et al., 2009; Cua and Stein, 2011; Kartal et al., 2012) and evidence of differential regulation of these gene sets in different genomic backgrounds has increased with every study. For instance, some studies of *Nitrosomonas europaea* ATCC 19718 report increased transcription of *amoCAB* genes and protein synthesis upon exposure to ammonium (Hyman and Arp, 1995; Sayavedra-Soto et al., 1996; Stein et al., 1997), whereas another study comparing expression levels between ammonium-starved and growing cells showed unvaried *amoCAB* transcript levels (Wei et al., 2006).

**Figure 1:**
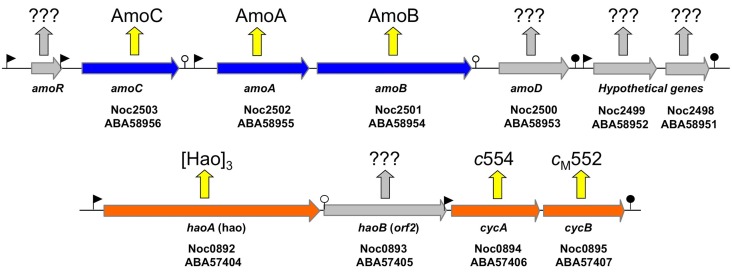
**Map of the *amo* gene cluster based on the genome sequence of *N. oceani* ATCC 19707**. Filled arrows represent *open reading frames*. The promoters and transcriptional terminators are represented by flags and stem loops, respectively. Week terminators are indicated by an open circle. The *amoR* gene (not annotated in the genome and thus not represented on the array) is unique to *Nitrosococcus oceani* (*strain ATTC 19707, AFC27, AFC132, and C-27*) and Noc2499 and Noc2498 are hypothetical genes conserved in the genome sequenced strains of all three species in the genus *Nitrosococcus (N. oceani, N. watsonii, and N. halophilus)*. Identified and hypothetical expression products are given by respective protein annotation or question marks, respectively.

According to the current literature, the gammaproteobacterial genus *Nitrosococcus* is exclusively represented by marine and high salt-tolerant AOB (O'Mullan and Ward, 2005; Campbell et al., 2011a). In contrast to genomes of betaproteobacterial AOB that encode multiple, nearly identical copies of *amoCAB* and *haoAB-cycAB* gene clusters, *Nitrosococcus* genomes encode only one copy of each (Klotz et al., 2006; Arp et al., 2007; Campbell et al., 2011a; Klotz and Stein, 2011). The *ncyA* gene exists as a single copy in all AOB genomes (Klotz and Stein, 2011). In contrast, the complement of quinol-oxidizing electron flow inventory encoded in the genomes of nitrosococci is more diverse than that encoded in betaproteobacterial AOB genomes (Klotz and Stein, 2011). Thus, *Nitrosococcus* is an ideal model for discriminating differences between signaling- and energy-mediated expression of genes encoding essential inventory for catabolism and chemosynthesis. Thus, far, it has been shown that ammonium induces expression of the *amoRCABD* gene cluster encoding the AMO complex (Figure 1), whereby the individual overlapping operons in this cluster exhibited different patterns of regulation and expression (El Sheikh and Klotz, 2008; El Sheikh et al., 2008).

In the present study, we used a Nimblegen-platform quadroplex microarray to investigate the differences between ammonium- and hydroxylamine-induced transcriptomes of *N. oceani* after denial of ammonia as an energy source. The premise for the experimental design was that genes similarly regulated between the ammonium and hydroxylamine treatments would be considered as “energy/redox status-regulated,” whereas genes more highly expressed under the ammonium compared to the hydroxylamine treatment would be considered as “ammonium-regulated.” The results allowed us to discriminate inventory that responds strongly and rapidly to a change in cellular redox status from exclusive members of the ammonia stimulon. The global transcriptional responses of *N. oceani* were compared to other AOB to better understand and define the core function and regulation of obligate ammonia-oxidizing chemolithotrophs.

## Materials and methods

### Bacterial growth and experimental treatments

*Nitrosococcus oceani* type-strain ATCC 19707 was obtained from the American Type Culture Collection and maintained at a temperature of 30°C in the dark without shaking on marine mineral salts medium as described previously (Graham et al., 2011). To attain sufficient biomass, multiple cultures were grown in 0.6- and 1-L batches of medium in 2- and 4-L Erlenmeyer flasks, respectively, and titrated to pH 8.0 daily with K_2_CO_3_. Cells were grown to late exponential growth phase as determined by NO^−^_2_ concentration, harvested by centrifugation (8000 × g, 15 min at 4°C), washed twice with NH_3_-free growth medium, and resuspended into 800 mL fresh NH_3_-free growth medium containing 5 mM HEPES. The resuspended cells were divided into four 200 mL aliquots for the following experimental treatments: (1) *NH^+^_4_-Starved*: Cells were denied energy and reductant by omitting (NH_4_)_2_SO_4_ from the growth medium for 24 h. (2) *NH^+^_4_-Induced*: Induction of gene expression by ammonium was investigated by exposing ammonium-starved cells (20 h) to 5 mM (NH_4_)_2_SO_4_ for 4 h. (3) *Control*: The control treatment was comprised of cells incubated in growth medium with 5 mM (NH_4_)_2_SO_4_ for 24 h. (4) *NH_2_OH - Induced*: Induction of gene expression by exposure to hydroxylamine was investigated by exposing ammonium-starved cells (23.5 h) to 0.2 mM NH_2_OH for 30 min. Biomass was collected by centrifugation following the specified exposure times and used immediately for RNA extraction.

### RNA extraction

RNA was extracted with the FastRNA Pro Blue kit (Qbiogene, Irvine, CA, USA) according to the manufacturer's protocol. RNA pellets were resuspended in 100 mL of nuclease-free 0.1 mM EDTA (Ambion, Austin, TX, USA). Resuspended RNA was checked for integrity by visualization of ribosomal bands on an Ethidium bromide-stained agarose gel and quantified by absorbance at 260 nm on a spectrophotometer (Beckman DU 640, Fullerton, CA, USA). Each extraction yielded 15–30 μg total RNA. Intact RNA samples were treated with RQ1 RNase-free DNase (Promega, Madison, WI, USA) according to the manufacturer's recommendations, ethanol-precipitated, and resuspended in 20 mL of 0.1 mM EDTA and re-checked for degradation. A portion of the RNA was used in cDNA synthesis and the remainder was stored at −20°C. RNA was converted to first-strand cDNA using 200 ng of random nonamer primer with Superscript III (Invitrogen, Carlsbad, CA, USA) reverse transcriptase according to the manufacturer's instructions at an extension temperature of 50°C for 60 min. A portion of the first-strand cDNA was used in second-strand cDNA synthesis according to the manufacturer's instructions. The second-strand cDNA was shipped to NimbleGen Systems (Reykjavik, Iceland) for processing, labeling and microarray hybridization.

### Array design, hybridization and analysis

The genome sequence from *N. oceani* (GenBank chromosome accession no. CP000127) was used by NimbleGen for microarray design and manufacture. The NimbleGen 4 × 72 K multiplex microarray slide contained 72,000 probe sets per array and four arrays per slide. Four replicates of the genome were included per array and six 60-mer probes represented each open reading frame (ORF) in the genome (the finished *N. oceani* genome is annotated to have 3017 ORFs). A quality control check (hybridization) was performed for each array, which contained on-chip control oligonucleotides. Double-stranded cDNA was random-prime labeled with Cy3-nonamers and hybridized to the microarrays. The microarrays were washed, dried, and scanned at 5 μ m resolution using a GenePix 4000B microarray scanner (Molecular Devices, Sunnyvale, CA). Data were extracted from scanned images using NimbleScan software (NimbleGen).

### Microarray data analysis

Investigation of reproducible differences between treatments was performed using the CLC Genomics Workbench software package. Data were processed using quantile normalization and background correction was performed using the robust multi-array average (RMA) method. Data were visualized with scatter plots to examine the distribution of hybridizing cDNAs to *Nitrosococcus* probes (equivalent to NGS reads). Intensities were adjusted to have the same interquartile range. A linear model fit was determined for each gene using the CLC Genomics Workbench.

Expression ratios were calculated from the averages of four independent microarray hybridizations (i.e., a total of 16 spots averaged per gene, per treatment) and presented as: (1) Induction of transcripts responsive to ammonium (NH^+^_4_-Induced/NH^+^_4_-Starved); (2) Induction of transcripts responsive to NH_2_OH (NH_2_OH-Induced/NH^+^_4_-Starved); (3) Repression or degradation of transcripts from denial of ammonium as an energy source (NH^+^_4_-Starved/Control); and (4) Transcripts responsive to ammonium in non-starved cells (NH^+^_4_-Induced/Control). Hybridization results, ratios, and standard deviations from replicate microarray hybridizations are provided in Supplemental Table 1.

## Results

### Gene expression ratios between energy-induced vs. starved cells

**Figure 2:**
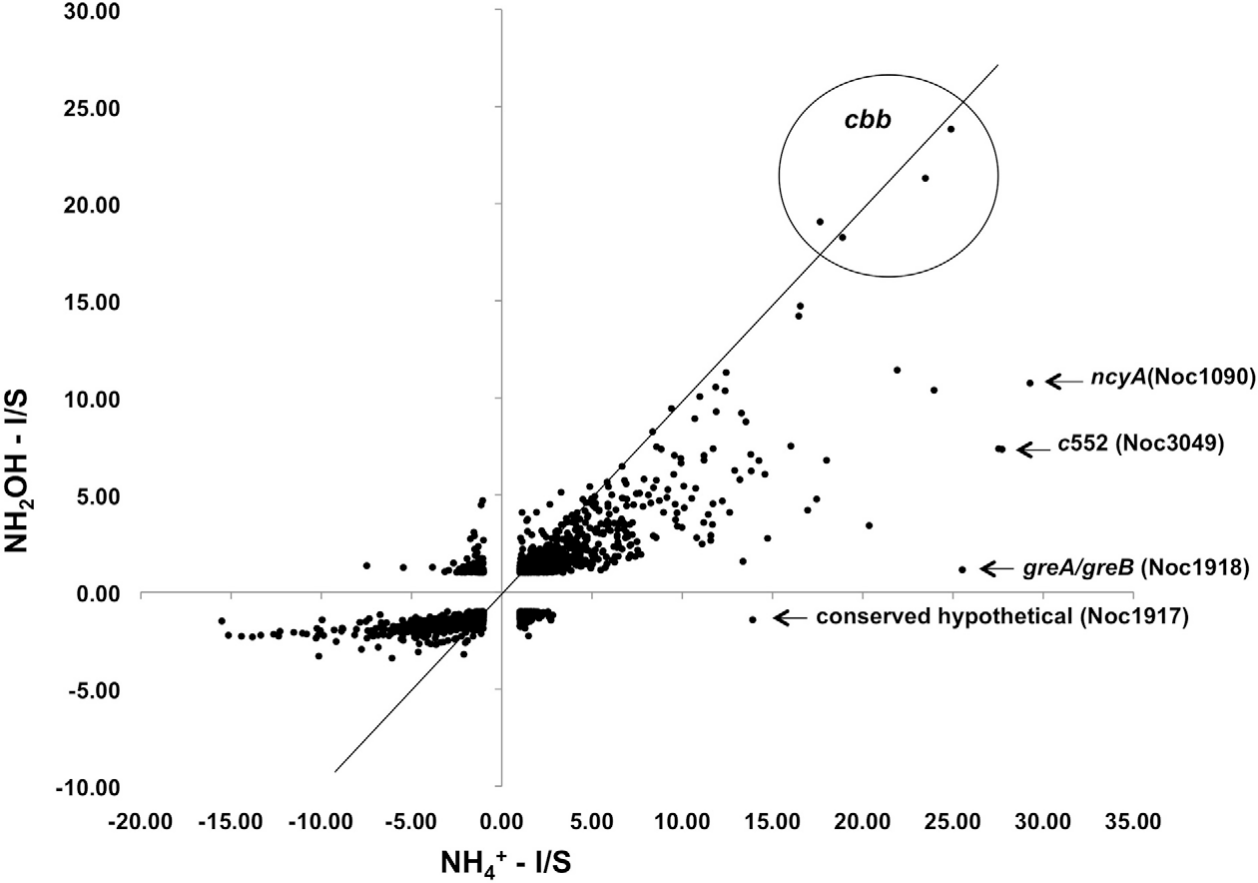
**Expression ratios for genes across the *N. oceani* genome (chromosome and plasmid) for hydroxylamine-induced versus ammonium-induced cells relative to a starved-cell control**.

Exposure of *N. oceani* cells to either ammonium or hydroxylamine as an energy source allowed examination of global gene expression as the result of a change in cellular redox status. Steady-state mRNA levels varied from +30 to −16-fold when provided with ammonium or hydroxylamine relative to cells deprived of energy (Figure 2). Responses to ammonium resulted in greater variation across the genome when compared to responses to hydroxylamine in genes that were under-expressed relative to starved control cells, however, variation in upregulated genes was similar between the two treatments (Figure 2).

Examination of individual genes and gene clusters affected by ammonium or hydroxylamine exposure showed the greatest response in modules for primary and secondary electron flow (Table 1) and carbon fixation (Figure 2; Table 2). It was expected that the primary inventory for chemolithotrophic ammonia oxidation (*amoCAB, haoAB, cycAB*, and *ncyA*), would be highly induced upon exposure to an energy-generating substrate, and particularly to ammonium. However, the steady-state levels of transcripts from ammonia monooxygenase (*amoCAB*, Noc2503, 2502, 2501) and hydroxylamine dehydrogenase (*haoA*, Noc0892) structural genes were not significantly changed, whereas the transcript levels of other genes in these two clusters (Noc2500, 2499; Noc0893, Noc0895) were significantly increased in response to ammonium or hydroxylamine and decreased in ammonium-starved relative to control cells (Figure 1; Table 1). Similarly, while levels of the *cycA* (Noc0894) transcript were not elevated, *cycB* (Noc0895) transcript levels were higher after exposure to ammonium or hydroxylamine. mRNA levels of the gene encoding nitrosocyanin (*ncyA*, Noc1090) were among the most elevated in response to ammonium or hydroxylamine (Figure 2; Table 1), and they were significantly reduced in ammonium-starved relative to control cells (Table 1). As for carbon fixation and assimilation, genes in the operon encoding Ribulose-1,5-bis-phosphate Carboxylase/Oxygenase (RuBisCO) was among the most highly expressed by ammonium or hydroxylamine as were genes in an operon encoding glycolytic enzymes likely involved in gluconeogenesis (Table 2). The gene encoding a key regulatory protein for N-assimilation, PII, was also highly expressed as a result of exposure to ammonium or hydroxylamine (Table 2).

**Table 1:** **Expression ratios of genes and gene clusters in catabolic modules for “Energy and Primary and Secondary Electron Flow**.”

**Locus tag**	**Gene product**	**Predicted function**	**Relative expression ratios**			
			**NH^+^_4_–I/S**	**NH_2_OH–I/S**	**S/C**	**NH^+^_4_–I/C**
Noc2503	AmoC	Ammonia monooxygenase subunit C	−1.11	−1.10	1.05	−1.06
Noc2502	AmoA	Ammonia monooxygenase subunit A	1.22 **(1.60)**	1.12 (11.5)	−1.32	−1.09
Noc2501	AmoB	Ammonia monooxygenase subunit B	1.41	1.22	−1.47	−1.04
Noc2500	AmoD	Periplasmic membrane protein (1 TMS)	**21.91**	**11.43**	**−10.91**	2.00
Noc2499	Hyp	Hypothetical periplasmic protein	**13.28**	**9.21**	**−5.39**	2.46
Noc2498	Hyp	Integral transmembrane protein (4TMS)	2.43	1.86	−1.56	1.56
Noc0089	NirK	Nitrite reductase nirK (EC:1.7.2.1)	−1.16 (10.10)	−1.17 (7.80)	−1.01	−1.17
Noc0889	Mco	Multi-copper oxidase	**6.12**	1.65	−2.34	2.64
Noc0890	CytL	Cytochrome c P460 (NO & NH2OH detox)	**6.80**	**5.75**	−2.62	2.59
(Noc0891)	GntR	Transcriptional regulator	1.88	1.15	1.15	1.73
Noc0892	HaoA	Hydroxylamine DH (EC:1.7.2.6, [HaoA]_3_)	3.88 (7.20)	3.61 (15.3)	−3.31	1.17
Noc0893	HaoB	Unknown [HaoA]_3_-associated protein	**10.97**	**10.07**	**−6.34**	1.73
Noc0894	CycA	Cytochrome c554	2.77	2.76	−2.28	1.21
Noc0895	CycB	Cytochrome cM552	**9.40**	**9.45**	−4.13	2.28
Noc0299	[Fe-S]	Rieske protein (CIII, EC:1.10.2.4)	**11.85 (13.10)**	**10.55** (49.60)	−4.39	2.70
Noc0298	CytB	Cytochrome b (CIII, EC:1.10.2.3)	4.00	3.66	−2.31	1.70
Noc0297	Cytc_1_	Cytochrome c_1_ (CIII, EC:1.10.2.2)	3.57	3.59	−2.15	1.65
Noc0552	CccA	Di-heme c552 (COG 2010; class IV)	**13.19**	**5.79**	−2.52	**5.22**
Noc0551	DsbA	DsbA oxidoreductase	**7.15**	2.44	−1.40	**5.10**
Noc0751	c552	Mono-heme c552 (COG 2863; class I)	**12.22** (3.90)	4.69 **(2.90)**	−2.18	**5.60**
Noc1089	Pgb	Protoglobin (heme binds O2, CO & NO)	**8.25**	4.58	−3.45	2.41
Noc1090	NcyA	Nitrosocyanin	**29.27**	**10.76**	**−14.23**	2.06
(Noc2967)	NsrR	Transcriptional regulator	2.32 **(1.50)**	1.64 **(2.10)**	−1.37	1.69
Noc2969	SenC	Nitric oxide reductase sNOR (coxBA-senC)	**8.54**	2.82	−1.64	**5.22**
Noc2970	NorY	Nitric oxide reductase sNOR (coxBA-senC)	2.74	2.00	−2.43	1.12
Noc2971	NorS	Nitric Oxide reductase sNOR (coxBA-senC)	**9.56**	**7.04**	**−7.02**	1.36
Noc3044	SU III	Type-A Complex IV HCO-1 (EC:1.9.3.1)	3.61	1.55	−2.85	1.27
Noc3045	SU IV	Type-A Complex IV HCO-1 (Assembly)	**6.93**	3.00	**−5.10**	1.36
Noc3046	SU I	Type-A Complex IV HCO-1 (EC:1.9.3.1)	**6.40**	2.93	**−5.16**	1.24
Noc3047	SU II	Type-A Complex IV HCO-1 (EC:1.9.3.1)	4.73 (9.70)	2.71 (5.00)	−4.51	1.05
Noc3048	Hyp	Cytoplasmic membrane protein (2 TMS)	**17.99**	**6.79**	**−11.79**	1.53
Noc3049	c552	Mono-heme c552 (COG 2863; class I)	**27.72**	**7.35**	**−12.61**	2.20
Noc3050	c552	Mono-heme c552 (COG 2863; class I)	**7.24** (17.10)	3.58 (7.00)	**−5.28**	1.37
Noc3073	AtpE	FoF1-Type ATP Synthase (EC:3.6.3.14)	**9.62**	3.73	−3.22	2.99
Noc3074	AtpB-F1	FoF1-Type ATP Synthase (EC:3.6.3.14)	**8.73**	4.71	−4.05	2.15
Noc3075	AtpC-F1	FoF1-Type ATP Synthase (EC:3.6.3.14)	**5.94**	3.56	−4.01	1.48
Noc3076	AtpA-F1	FoF1-Type ATP Synthase (EC:3.6.3.14)	**11.70**	4.55	−**5.17**	2.27
Noc3077	AtpD-F1	FoF1-Type ATP Synthase (EC:3.6.3.14)	**7.08**	3.47	−4.63	1.53
Noc3078	AtpB-F0	FoF1-Type ATP Synthase (EC:3.6.3.14)	**17.44**	4.79	−**5.68**	3.07
Noc3079	AtpC-F0	FoF1-Type ATP Synthase (EC:3.6.3.14)	3.76	2.26	−3.05	1.23
Noc3080	AtpA-F0	FoF1-Type ATP Synthase (EC:3.6.3.14)	1.61	1.20	−1.48	1.08
Noc2552	NuoN	NDH-1 (Reverse Complex-I)	1.34	−1.39	−1.15	1.17
Noc2553	NuoM	NDH-1 (Reverse Complex-I)	−1.26	−1.71	1.03	−1.22
Noc2554	NuoL	NDH-1 (Reverse Complex-I)	1.49	−1.35	−1.04	1.43
Noc2555	NuoK	NDH-1 (Reverse Complex-I)	3.17	1.09	−1.15	2.74
Noc2556	NuoJ	NDH-1 (Reverse Complex-I)	3.75	1.51	−1.48	2.54
Noc2557	NuoI	NDH-1 (Reverse Complex-I)	3.33	1.46	−1.20	2.76
Noc2558	NuoH	NDH-1 (Reverse Complex-I)	−1.12	−1.18	1.05	−1.07
Noc2559	NuoG	NDH-1 (Reverse Complex-I)	2.66	1.32	−1.07	2.47
Noc2560	NuoF	NDH-1 (Reverse Complex-I)	1.57	1.36	−1.12	1.40
Noc2561	NuoE	NDH-1 (Reverse Complex-I)	**5.35**	1.69	−1.28	4.19
Noc2562	NuoD	NDH-1 (Reverse Complex-I)	2.07	1.33	−1.28	1.62
Noc2563	NuoC	NDH-1 (Reverse Complex-I)	3.54	1.94	−1.59	2.23
Noc2564	NuoB	NDH-1 (Reverse Complex-I)	2.58	1.38	−1.25	2.07
Noc2565	NuoA	NDH-1 (Reverse Complex-I)	4.15	1.38	−1.28	3.25
Noc0474	NuoA	NuoAHJKLLMN (C I-membrane arm)	−1.89	−1.45	1.09	−1.73
Noc0475	NuoH	NuoAHJKLLMN (C I-membrane arm)	**−12.29**	−2.02	1.23	**−9.98**
Noc0476	NuoJ	NuoAHJKLLMN (C I-membrane arm)	**−8.88**	−2.01	1.29	**−6.90**
Noc0477	NuoK	NuoAHJKLLMN (C I-membrane arm)	−3.39	−1.43	1.23	−2.75
Noc0478	NuoL	NuoAHJKLLMN (C I-membrane arm)	**−10.05**	−1.99	1.34	**−7.48**
Noc0479	NuoL	NuoAHJKLLMN (C I-membrane arm)	**−11.52**	−2.08	1.39	**−8.27**
Noc0480	NuoM	NuoAHJKLLMN (C I-membrane arm)	**−5.23**	−2.06	1.23	−4.24
Noc0481	NuoN	NuoAHJKLLMN (C I-membrane arm)	−3.50	−1.71	1.20	−2.93
Noc1797	Cyt_C	Mono-heme cytochrome *c* protein	**−7.34**	−1.37	**9.24**	1.25
Noc1798	Cyt_C	Mono-heme cytochrome *c* protein	**−7.52**	−1.51	**6.86**	−1.10
Noc1799	NemA	NADH-flavin oxidoreductase (EC:1.3.1.34)	**−9.95**	−1.43	**12.43**	1.25
Noc1800	Cyt_C	Di-heme cytochrome c protein	**−15.51**	−1.49	**13.70**	−1.13

Values represent average ratios from four hybridization experiments per gene, per treatment. Bolded ratios indicate those that are considered to have significant differences (changes greater than 5-fold) in gene expression between compared treatments. Values in parentheses represent ratios from qPCR experiments reported previously (Graham et al., 2011) employing the same RNA preparations as used for the microarray hybridizations. Values in bold indicate that both methods yielded consistent results.

**Table 2:** **Expression ratios of genes and gene clusters in catabolic modules for “Carbon Fixation and Assimilation” and “Nitrogen Assimilation**.”

**Locus tag**	**Gene product**	**Predicted function**	**Relative expression ratios**			
			**NH^+^_4_–I/S**	**NH_2_OH–I/S**	**S/C**	**NH^+^_4_–I/C**
Noc0330	Hyp	Associated with RuBisCO operon	**17.64**	**19.07**	−2.84	**6.20**
Noc0331	CbbX	RuBisCO (ATPase)	**24.89**	**23.83**	−4.38	**5.69**
Noc0332	CbbS	RuBisCO (small subunit)	**18.88**	**18.26**	**−11.07**	1.71
Noc0333	CbbL	RuBisCO (large subunit)	**23.47**	**21.31**	**−12.45**	1.88
Noc2808	Tkt2	Transketolase (EC:2.2.1.1)	**11.20**	**6.79**	−4.51	2.49
Noc2807	GapA	Glyceraldehyde-3-P dehydrogenase (EC:1.2.1.12)	**7.88**	**5.81**	−4.70	1.68
Noc2806	Pgk	Phosphoglycerate kinase (EC:2.7.2.3)	2.74	2.31	−2.18	1.25
Noc2805	PykF	Pyruvate kinase (EC:2.7.1.40)	2.17	1.62	−1.40	1.55
Noc2804	ALDOB	Fructose-bis-phosfate aldolase (EC:4.1.2.13)	**5.88**	4.54	−3.12	1.89
Noc2492	RPE	Ribulose-phosfate 3-epimerase (EC:5.1.3.1)	**9.53**	**6.06**	−2.02	4.73
Noc2493	PGLP	Phosphoglycolate phosphatase (EC:3.1.3.18)	2.59	2.43	−1.57	1.65
Noc0715	GlnB/K	Nitrogen regulatory protein P-II	**13.82**	**6.23**	−2.28	**6.05**
Noc2573	CPS	Carbamoyl-phosfate synthase, small subunit	**6.97**	2.84	−1.51	4.63
Noc2572	CPS	Carbamoyl-phoshate synthase, large subunit	**4.96**	1.56	−1.39	3.59

Values represent average ratios from four hybridization experiments per gene, per treatment. Bolded ratios indicate those that are considered to have significant differences in gene expression between compared treatments (changes greater than 5-fold).

Other noteworthy catabolic inventory induced by ammonium or hydroxylamine included genes encoding cytochrome P460 (*cycL*, Noc0890), mono- (Noc3049) and di-heme cytochrome *c*552 proteins (Noc0552), Rieske protein of Complex III (Noc0299), and the NorS component (Noce2971) of cytochrome *c*-linked nitric oxide reductase, sNOR (Table 1). Stress tolerance genes encoding tellurium resistance protein TerB (Noc1001) and the protein chaperone GroES (Noc2922), and a gene encoding the acyl carrier protein AcpP (Noc1664) were also among the most highly induced by exposure to ammonium or hydroxylamine (Table 3).

**Table 3:** **Expression ratios of genes and gene clusters in other functional categories**.

**Locus tag**	**Gene product**	**Predicted function**	**Relative expression ratios**			
			**NH^+^_4_–I/S**	**NH_2_OH–I/S**	**S/C**	**NH^+^_4_–I/C**
**DNA**						
Noc0448	HsdR	Type I site-specific deoxyribonuclease	**−11.04**	−2.12	1.30	**−8.51**
Noc0447	HsdR	Type I restriction-modification system endonuclease	−1.27	−1.86	1.28	1.01
Noc0446	HsdS	Restriction endonuclease S subunit	**−10.81**	−2.16	1.21	**−8.92**
Noc1205	HsdR	Type III restriction enzyme	−1.67	−1.31	1.20	−1.38
Noc1204	HsdS	Restriction modification system DNA specificity domain	−3.72	−1.74	1.19	−3.11
Noc1203	PnpA	DNA polymerase, beta-like region	**−8.15**	−2.21	1.42	**−5.74**
Noc1202	DUF86	Hypothetical	**−10.29**	−2.38	1.49	**−6.91**
Noc1201	YfiC	N-6 DNA methylase	**−5.04**	−1.77	1.21	−4.16
Noc2535	PnpA	DNA polymerase, beta-like region	**−8.05**	−1.92	1.21	**−6.67**
Noc2536	PnpB	Nucleotidyltransferase substrate binding protein	**−5.35**	−1.43	1.19	−4.48
**TRANSCRIPTION**						
Noc1918	GreA/B	Transcription elongation factor	**25.52**	1.15	**−20.84**	1.22
Noc1917	Hyp	Hypothetical protein	**13.90**	−1.67	**−11.56**	1.20
Noc2610	RNP-1	RNA binding protein	**16.95**	4.22	−2.27	**7.46**
Noc2010	DksA	C-4 type zinc finger protein	**16.02**	**7.52**	−2.16	**7.43**
**TRANSLATION**						
Noc2332	RplL	Large subunit ribosomal protein L7/L12	**23.95**	**10.40**	**−5.74**	4.17
Noc2640	RpmB	Large subunit ribosomal protein L28	**16.45**	**14.21**	**−6.57**	2.51
Noc2641	RpmG	Large subunit ribosomal protein L33	**12.43**	**11.30**	**−5.51**	2.28
Noc2309	RplF	Large subunit ribosomal protein L6	**11.44**	3.99	**−5.10**	2.24
Noc3037	RpsT	Small subunit ribosomal protein S20	**11.18**	3.58	−1.63	**6.86**
Noc2319	RplV	Large subunit ribosomal protein L22	**10.69**	**8.93**	**−5.04**	2.13
Noc2122	YhbC	Ribosomal maturation protein	**9.71**	4.08	−1.76	**5.51**
Noc0042	RpsU	Small subunit ribosomal protein S21	**5.74**	1.26	−1.14	**5.04**
**FLAGELLA/PILI**						
Noc0833	MotA	Proton channel	−3.24	−1.77	1.22	−2.66
Noc0834	MotB	Flagellar motor protein MotB	**−6.70**	−1.77	1.19	**−5.62**
Noc2365	FliD	Flagellar hook-associated protein 2	**−7.15**	−2.09	1.40	**−5.14**
Noc1658	FliH	Flagellar assembly	**7.71**	1.93	−1.37	**5.64**
Noc2270	FimT	Type-IV fimbiral pilin related signal peptide protein	**−10.24**	−1.88	1.24	**−8.24**
Noc2271	PilV	Type IV pilus assembly protein	**−6.18**	−1.96	1.41	−4.39
Noc2272	PilW	Type IV pilus assembly protein	**−5.49**	−2.28	1.61	−3.40
Noc2273	PilX	Type IV pilus assembly protein	−3.33	−1.79	1.50	−2.23
Noc2274	PilY	Type IV pilus assembly protein PilY1	**−6.61**	−1.97	1.26	**−5.25**
Noc2275	PilE	Bacterial general secretion pathway protein H	−2.78	−1.48	1.27	−2.18
**TRANSPORT**						
Noc1626	Sul1	Sulfate transporter	**20.35**	3.42	**−16.51**	1.24
Noc1194	Fur	Ferric uptake regulation protein	**6.29**	1.84	−1.17	**5.35**
Noc0294	CzcA	Heavy metal efflux pump; CzcA family	**−6.53**	−1.89	1.17	**−5.54**
**STRESS TOLERANCE**						
Noc1001	TerB	Tellurium resistance	**16.54**	**14.73**	**−6.64**	2.50
Noc2922	GroES	Protein folding chaperone	**14.25**	**6.77**	−2.68	**5.30**
**LIPIDS**						
Noc1664	AcpP	Acyl carrier protein; fatty acid biosynthesis	**27.53**	**7.38**	**−5.36**	**5.13**
Noc0157	HlyIII	Channel protein, hemolysin III family COG1272	**−13.35**	−2.22	1.36	**−9.83**

Values represent average ratios from four hybridization experiments per gene, per treatment. Bolded ratios indicate those that are considered to have significant differences in gene expression between compared treatments (changes greater than 5-fold).

Data from RT-qPCR assays presented in Table 1 were performed using the same RNA preparations as for microarray hybridizations, employing primer sets designed against core catabolic genes as reported elsewhere (Graham et al., 2011). Only few of the ratios derived from RT-qPCR matched those from microarray hybridization experiments; for instance, genes encoding *haoA* (Noc0892), *nirK* nitrite reductase (Noc0089) and two genes in the Complex IV terminal oxidase gene cluster (Noc3047, 3050) were highly expressed according to RT-qPCR in cells exposed to ammonium or hydroxylamine, but were not highly expressed according to microarray hybridization. Also surprising was high expression of *amoA* upon exposure to hydroxylamine, but not to ammonium, according to RT-qPCR, but not microarray hybridization experiments. The pattern of *amoA* expression using arrays is also in contradiction to prior studies on regulation of the ammonia monooxygenase operon by ammonium in *N. oceani*, which was assessed by RT-qPCR and Northern analysis (El Sheikh and Klotz, 2008; El Sheikh et al., 2008).

Significantly reduced steady-state transcript levels were identified only when comparing results from ammonium-exposed and starved cells, indicating transcript degradation or gene repression as part of the ammonium-responsive stimulon rather than as a response to a change in cellular redox status.

### Genes responsive to ammonium as a signaling molecule

Genes for which significant changes in transcript levels were identified after exposure to ammonium, but not to hydroxylamine, relative to both starved and non-starved control cells can be considered *bona fide* components of the ammonia-responsive stimulon. Such genes with increased transcript levels included those encoding DsbA disulfide oxidoreductase (Noc0551), a mono-heme cytochrome *c*552 (Noc0751), the SenC component of sNOR nitric oxide reductase (Noc2969), two transcriptional regulators (Noc2610, 2010), ribosomal structural proteins (Noc3037, 2122, 0042), flagellar assembly protein FliH (Noc1658), and a ferric uptake regulatory protein (Noc1194) (Tables 1, 3). Genes with decreased transcript levels included several that contribute to synthesis of forward Complex I (Noc0475-0476, 0478-0479), restriction endonucleases and DNA polymerases (Noc0448, 0446, 1203, 2535), flagellar and Type IV pilus proteins (Noc0834, 2365, 2270-2272, 2274), a Czc heavy metal efflux pump (Noc0294), and a hemolysin channel protein (Noc0157) (Tables 1, 3).

A number of genes also showed significant changes in transcript levels upon exposure to ammonium, but not hydroxylamine, relative to the ammonium-starved, but not to the control cells taken directly from batch cultures. Highly expressed genes in this category included those encoding a multi-copper oxidase (Noc0889), clusters encoding Complex IV terminal oxidase (Noc3045, 3036, 3050), F_0_F_1_ ATPase (Noc3073-3078) and carbamoyl-phosfate synthase (Noc2573-2572), and a very highly expressed sulfate transporter (Noc1626) (Tables 1–3). Genes with reduced transcript levels in this category included a four-gene cluster encoding cytochrome *c* related proteins (Noc1797-1800).

### Degradation of transcripts during starvation from ammonium

Transcript degradation was assessed by comparing expression of genes in ammonium-starved cells relative to non-starved control cells. The most depleted transcripts in starved cells included those encoding the *amoD* transcript (member of the *amo* operon, Noc2500), the *haoB* transcript (member of the *hao* operon, Noc0893), *ncyA* (nitrosocyanin, Noc1090), the *norS* transcript (member of the nitric oxide reductase operon, Noc2971), several genes in the Complex IV terminal oxidase gene cluster (Noc3045-3050), transcripts encoding CbbL and CbbS of RuBisCO (Noc0332-0333), transcriptional elongation factors (Noc1918-1917), a number of ribosomal proteins (Noc2332, 2640, 2641, 2309, 2319), the ammonium-induced sulfate transporter (Noc1626), the TerB tellurium resistance protein (Noc1001), and acyl carrier protein AcpP (Noc1664) (Tables 1–3). Only the four-gene cluster encoding cytochrome *c* related proteins with unknown function was represented with higher transcript levels in starved relative to non-starved cells (Noc1797-1800) (Table 1).

## Discussion

The primary findings from this study are that: (1) *N. oceani* was far more responsive to general changes in redox/energy status than to ammonium as a signaling molecule alone, (2) genes involved in carbon fixation were among the most highly affected by a change in redox/energy status, which makes sense given that this is the most energy and reductant requiring process in chemolithotrophic metabolism; (3) several genes involved in primary catabolic electron flow were regulated by changes in redox/energy status, however, not all clustered or operonal genes were represented by equal and/or equal changes in transcript levels including those genes shown to be regulated by ammonium in prior studies (Sayavedra-Soto et al., 1996; El Sheikh and Klotz, 2008; El Sheikh et al., 2008); and (4) transcription of *ncyA* (nitrosocyanin) was the most highly responsive to changes in redox/energy status, indicating an essential function to bacterial ammonia chemolithotrophy. Patterns of transcript abundance in *N. oceani* were significantly different from those observed in certain marine metatranscriptomes where *amoA, amtB* (ammonium transporter), and *nirK* (nitrite reductase) mRNAs from ammonia-oxidizing thaumarchaeota were among the most highly represented (Gifford et al., 2010). This perhaps indicates a different response to redox/energy status between the marine ammonia-oxidizing bacteria and thaumarchaeota. Furthermore, the results suggest that key inventory and/or its principle regulation may be entirely different between representatives of ammonia-oxidizing chemolithotrophs from two domains of life.

Several genes encoding primary catabolic and carbon fixation inventory were responsive to ammonium or hydroxylamine and also exhibited reduced expression during ammonium-starvation. This suggests a direct linkage between energy homeostasis—the provisional usage of catabolic energy and reducing power—with carbon fixation and oxidative phosphorylation. The role of nitrosocyanin in catalysis and electron transfer has been discussed controversially in the literature without defining its role in ammonia chemolithotrophy other than its exclusivity to obligate ammonia-oxidizing bacteria (Klotz and Stein, 2011). The null hypothesis to disprove in the future is that nitrosocyanin determines whether electrons extracted by HAO from hydroxylamine will reduce either cytochromes *c*554 or *c*M552. The specific electron acceptor will then determine whether electrons directly enter the quinone pool or whether they become available for distribution to other electron sinks such as sNOR. Given that two electrons can contribute to energy conservation per ammonia molecule oxidized, complemented by the many possible electrogenic and electroneutral electron flow processes in the branched oxidative electron transport chain of *Nitrosococcus* (Klotz and Stein, 2011; Simon and Klotz, 2013), the need for controlling the direction of electron flow is evident. Increased expression of genes encoding components of AMO, HAO, CytB, cytochrome P460, sNOR, and nitrosocyanin with either ammonium or hydroxylamine suggests that oxidative electron flow favors not only nitrite production but, in particular the removal of nitric oxide by sNOR (reduction to N_2_O) or cytochrome P460 (oxidation to nitrite) when NO is produced by oxidation of hydroxylamine (Stein, 2011). This activity makes sense for maximizing electron flow between AMO/HAO with Complexes III and IV at high substrate oxidation rates by using periplasmic cytochromes and nitric oxide reductase as catabolic overflow electron sinks (Stein, 2011).

Although the ammonia stimulon as revealed from the microarray data was sparsely populated compared to the gene complement regulated by redox/energy status, it can be concluded that increased transcript availability of genes encoding Complex IV and ATPase along with decreased transcript availability of genes encoding forward Complex I suggests a boost to the production of proton motive force and ATP. This result is consistent with the maximization of catabolic electron flow and upregulation of periplasmic electron sinks to optimize electron flow to the terminal oxidase (Stein, 2011).

One caveat of the results presented in Table 1 is the lack of consistency between the data sets generated with the microarray approach and in the RT-qPCR experiments. Although neither method revealed increased steady-state levels of *amoA* mRNA following exposure to ammonium, *amoA* mRNA levels following exposure to hydroxylamine and *haoA* and *nirK* transcript levels following exposure to either ammonium or hydroxylamine were significantly higher when using RT-qPCR compared to employing the microarray approach. It is possible that that the identified differences in both data sets are due to the inherent differences in hybridization kinetics between the two technologies, differential processing that occurs at least in *amo* (El Sheikh and Klotz, 2008) and *hao* (Poret-Peterson et al., 2008) transcripts, and differences in location of hybridizing regions across each gene. It is also important to realize that the analysis of both raw data sets utilized different normalization procedures.

The differences observed between techniques suggest that a quantitation of differences in transcriptional regulation and processing should be based on a gene-by-gene analysis using linearized standard templates in order to reveal why transcripts of certain members of gene clusters and operons are being detected at higher copy numbers than others across a microarray. Regardless of which technique is more accurate, when comparing gene expression data in *N. oceani* with that from betaproteobacterial ammonia-oxidizers (Bollmann et al., 2005; Wei et al., 2006; Cua and Stein, 2011; Kartal et al., 2012), it is evident that ammonia-dependent chemolithotrophy does not follow a single regulatory principle. While each phylotype appears to have maintained regulatory principles tied to the history of a given genome, they appear to have also adapted slightly different regulatory features that they acquired together with cohort-specific inventory to maximize their survival and growth, a process that likely also varied based on the environment (i.e., marine vs. freshwater and soil).

Taken together, this study effectively separated the effects of ammonium in its unprotonated form (ammonia) as an energy source from its role as a signaling molecule on regulatory processes in *N. oceani* that reflect themselves in varying steady-state transcript levels. The experimentally forced changes to cellular redox/energy status of *N. oceani* cells exerted a strong effect on inventory implicated in carbon fixation and catabolic electron flow to the terminal oxidase, indicating that these two functions are the most significant to survival and growth of this bacterium. It has been shown before that minuscule concentrations of ammonium are sufficient for the induction of gene expression (El Sheikh and Klotz, 2008; El Sheikh et al., 2008). In the marine environment, obligate ammonia-oxidizing bacteria are likely not thwarted by access to ammonium, but rather by environmental changes that could result in misdirection of electron flow along the catabolic pipeline. The identified coordinated expression of catabolic inventory, periplasmic electron sinks, nitrosocyanin and carbon fixation genes thus suggests that *N. oceani* is well adapted to quickly sense changes in energy availability to optimize growth and survival in a changing environment.

### Conflict of interest statement

The authors declare that the research was conducted in the absence of any commercial or financial relationships that could be construed as a potential conflict of interest.
